# Measurement‐guided volumetric dose reconstruction for helical tomotherapy

**DOI:** 10.1120/jacmp.v16i2.5298

**Published:** 2015-03-08

**Authors:** Cassandra Stambaugh, Benjamin Nelms, Theresa Wolf, Richard Mueller, Mark Geurts, Daniel Opp, Geoffrey Zhang, Eduardo Moros, Vladimir Feygelman

**Affiliations:** ^1^ Department of Physics University of South Florida Tampa FL USA; ^2^ Canis Lupus LLC Merrimac WI USA; ^3^ Live Oak Technologies LLC St. Louis MO USA; ^4^ Department of Radiation Oncology Moffitt Cancer Center Tampa FL USA; ^5^ Department of Human Oncology University of Wisconsin Madison WI USA

**Keywords:** helical tomotherapy QA, diode array, measurement‐guided dose reconstruction

## Abstract

It was previously demonstrated that dose delivered by a conventional linear accelerator using IMRT or VMAT can be reconstructed — on patient or phantom datasets — using helical diode array measurements and a technique called planned dose perturbation (PDP). This allows meaningful and intuitive analysis of the agreement between the planned and delivered dose, including direct comparison of the dose‐volume histograms. While conceptually similar to modulated arc techniques, helical tomotherapy introduces significant challenges to the PDP formalism, arising primarily from TomoTherapy delivery dynamics. The temporal characteristics of the delivery are of the same order or shorter than the dosimeter's update interval (50 ms). Additionally, the prevalence of often small and complex segments, particularly with the 1 cm Y jaw setting, lead to challenges related to detector spacing. Here, we present and test a novel method of tomotherapy‐PDP (TPDP) designed to meet these challenges. One of the novel techniques introduced for TPDP is organization of the subbeams into larger subunits called sectors, which assures more robust synchronization of the measurement and delivery dynamics. Another important change is the optional application of a correction based on ion chamber (IC) measurements in the phantom. The TPDP method was validated by direct comparisons to the IC and an independent, biplanar diode array dosimeter previously evaluated for tomotherapy delivery quality assurance. Nineteen plans with varying complexity were analyzed for the 2.5 cm tomotherapy jaw setting and 18 for the 1 cm opening. The dose differences between the TPDP and IC were 1.0%±1.1% and 1.1%±1.1%, for 2.5 and 1.0 cm jaw plans, respectively. Gamma analysis agreement rates between TPDP and the independent array were: 99.1%±1.8% (using 3% global normalization/3 mm criteria) and 93.4%±7.1% (using 2% global/2 mm) for the 2.5 cm jaw plans; for 1 cm plans, they were 95.2%±6.7% (3% G/3) and 83.8%±12% (2% G/2). We conclude that TPDP is capable of volumetric dose reconstruction with acceptable accuracy. However, the challenges of fast tomotherapy delivery dynamics make TPDP less precise than the IMRT/VMAT PDP version, particularly for the 1 cm jaw setting.

PACS number: 87.55Qr

## I. INTRODUCTION

As treatment plans become more complex, the need for QA increases. Therefore, it is a current standard of practice to perform a patient‐specific, end‐to‐end test (more colloquially known as per‐patient QA) for each intensity‐modulated radiation therapy case, which includes helical tomotherapy.^(1)^ The agreement between the measured and planned dose distributions is typically quantified by some combination of the percent dose error and distance‐to‐agreement (DTA) criteria or by gamma index (γ)‐analysis.^(2)^ While reliable agreement between the calculated and measured/reconstructed dose in a geometrical phantom is the basis for the dosimetric commissioning of an intensity‐modulated radiotherapy (IMRT) system, its value for the meaningful patient‐specific, end‐to‐end testing is less clear. Gamma analysis passing rates for either per‐beam single‐plane^(3)^ or quasi‐3D^(4)^ array geometries, have weak (and sometimes counter‐intuitive) correlation with the conventional clinical dose‐volume histogram (DVH) metrics. On the other hand, direct comparison of the planned and deliverable DVHs exhibits higher sensitivity and specificity, and is expected to be more clinically meaningful and intuitive to both the physician and the physicist.^4^, ^5^, ^6^, ^7^ One of the published methods for measurement‐guided dose reconstruction (MGDR) on a patient dataset involves a measurement with a helical diode array, ArcCHECK (AC) and subsequent processing by 3DVH software (Sun Nuclear Corp., Melbourne, FL).^(8)^ The method was further validated for volumetric‐modulated arc therapy (VMAT) against different dosimeters in both homogeneous^9^, ^10^ and heterogeneous anthropomorphic (thoracic)^(11)^ phantoms. Tomotherapy patient‐specific QA could equally benefit from the volumetric reconstruction of the deliverable patient dose. While the general approach remains similar to VMAT, the unique characteristics of the TomoTherapy Hi·Art treatment system (Accuray Inc., Madison, WI) dose delivery required substantial, conceptual modifications of the algorithm. In this paper, we describe this novel tomotherapy dose reconstruction method and validate it by sampling the produced 3D dose and comparing to independent measurements by an ion chamber and a biplanar array dosimeter.^12^, ^13^, ^14^

## II. MATERIALS AND METHODS

### A. Measurement‐guided dose reconstruction (MGDR)

#### A.1 The challenges of tomotherapy

**Figure 1:**
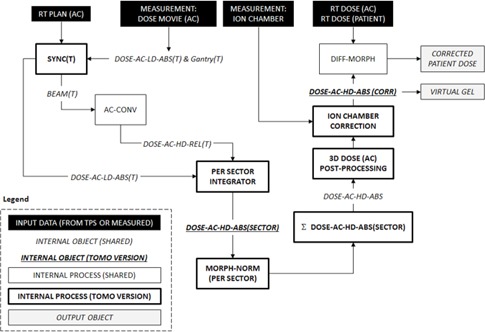
The workflow diagram of the Tomo‐PDP process. The novel steps specific to TPDP are in bold. The RT Plan is first synchronized to the measurement (SYNC[T]) and the calculated time‐resolved subbeams are grouped into sectors (PER SECTOR INTEGRATION). The sector doses are then morphed based on the measured entry and exit doses (MORPH‐NORM [PER SECTOR]). These morphed dose grids are summed to give the cumulative 3D phantom dose (E DOSE‐AC‐HD‐ABS [SECTOR]). Ion chamber measurements can be included to further correct the reconstructed dose. The resulting high‐density dose grid can be used for dose comparisons on the phantom (VIRTUAL GEL) or further processed to produce measurement‐guided dose reconstruction on the patent dataset. See Methods A.2 for detailed explanations of each step of the process.

The MGDR method applicable to conventional (C‐arm) linear accelerators is called ArcCHECK Planned Dose Perturbation (ACPDP). Helical tomotherapy presents many unique challenges that require a new variation of the ACPDP algorithm, which we will call Tomo‐PDP (TPDP). Figure 1 summarizes the workflow of the TPDP model to compare and contrast with a similar workflow diagram already presented for ACPDP.^(8)^ Some of the major MGDR challenges unique to helical tomotherapy are listed below.

##### A.1.1 Delivery dynamics (gantry)

ACPDP reconstructs the dose for time‐discretized subbeams. A gantry rotation period for VMAT beams on a conventional linac is no less than 60 s. ACPDP divides a VMAT beam into subbeams based on a gantry change threshold of $∼2^{\circ }$ and, as a result, the typical ACPDP subbeam spans on the order of 0.3 s. The AC measurement time resolution is 0.05 s, so a subbeam is certain to include multiple measurement updates. On the other hand, tomotherapy gantry rotation speed is much faster — up to five rotations/min, or a period of 12 s (30°/s). A subbeam covering 2° thus spans less than 0.07 s, which is on the order of a single AC measurement update interval. Therefore, the gantry speed presents challenges for dose reconstruction and causes sensitivities to even the smallest errors in derived or simulated gantry angle compared to actual.

##### A.1.2 Delivery dynamics (MLC leaves)

Tomotherapy delivery is based on 51 intervals per rotation, spanning about 7.06°. During rotation through each interval, the leaves snap open and then closed symmetrically about the middle of the interval. The time they stay open depends on their “open fraction”, producing a “strobe” effect in dose delivery. The 7° interval span can happen as fast as 0.235 s, and for small open fractions the leaf open time can approach as low as 0.03 s. That is just a fraction of the measurement update period, thus presenting another challenge unique to the delivery dynamics in relation to the measurement time resolution.

##### A.1.3 Sub‐beam size and complexity

Tomotherapy MLC leaves move in the IEC Y direction and are considered binary, though naturally there is some latency as the leaves open and close. The leaf widths are 6.25 mm at the isocenter, and the allowed field lengths (Y jaw settings) are approximately 1, 2.5, and 5 cm. Given the leaf dynamics and the strobe effect of all leaves being forced closed 51 times per rotation, at any given time the irradiated field can be very complex, consisting of small beamlets which are often discontiguous. The detector separation in the AC phantom is 10 mm along and across the diode helix.^(15)^ As a result, some tomotherapy subbeams intersect few diodes at entry and exit, thus limiting the capability of measurement guidance if subbeams are reconstructed as in conventional ACPDP (i.e., as a function of very small times corresponding to threshold geometry changes). This effect becomes more pronounced as the jaw setting decreases.

##### A.1.4 Beam parameters in DICOM RT Plan

The tomotherapy MLC dynamics are not specified in the RT Plan as leaf positions over a number of control points as with IMRT or VMAT. Instead, 51 control points per rotation are provided, but they do not explicitly define any MLC leaf positions; rather, a “sinogram” of the per‐leaf open fractions is defined per control point, as a private DICOM tag. From the open fractions, the opening and closing times of each leaf are derived for each of the 51 intervals per rotation. If there is nonnegligible leaf latency, or device‐specific MLC calibration/air pressure variation, it is not reflected in the RT Plan object.

##### A.1.5 Synchronization of time‐resolved measurements to beam parameters

**Figure 2:**
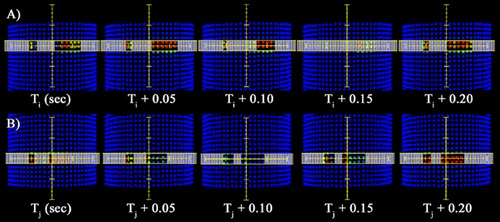
In this figure, the top and bottom row show two different 0.25 s intervals of MLC leaf arrangements vs. the entry diode doses (as a function of time) measured by the 3D dosimeter. Both 0.25 s time intervals are from the same irradiation of the TG‐119 head and neck plan. In each row, the entry diode doses accumulated in each 0.05 s subinterval are shown projected behind the nearest MLC open/closed positions derived directly from the sinogram data in the DICOM RT Plan. The sinogram and 4D delivery have been optimally synchronized in time. For row (A), all 0.05 second subintervals seem well‐matched (MLC openings vs. diode dose exposure) except for the Ti+0.15 s subinterval where according to the sinogram all MLCs should be shut, while clearly the diodes are receiving dose during this time. The seemingly out‐of‐sync subinterval is evidence of latent dose (from shutting leaves from prior control point) or leading dose (from opening leaves from the next control point) that results when absolute synchronization is not possible due to deviations in actual MLC positions from the expected positions defined by the sinogram data and control point time stamps. Row (B) illustrates a different kind of temporal challenge from the same irradiation. The pattern of nominal MLC arrangements vs. measured entry dose values suggests a 0.15 s time misalignment, which could be caused by actual delivery dynamics that do not match nominal as exported by the TPS in the RT Plan.

ACPDP relies on an accurate virtual inclinometer^(15)^ and robust synchronization logic, called “Sync(T) ” in Fig. 1, to assign absolute (accelerator) times to each control point in the RT plan, which are required when processing each subbeam. However, for tomotherapy, given the dearth of diodes irradiated at any given short time period and the high gantry speed, the AC virtual inclinometer cannot reliably derive actual gantry angle as a function of time from the AC signals. Instead, gantry angle vs. time must be taken directly from the RT Plan object, which is possible because tomotherapy's cumulative metersets are based on irradiation time (min) rather than monitor units. However, this causes a reliance on the coincidence of the RT Plan's gantry angle vs. time relationship with the actual delivery, which turns out to be not sufficiently accurate at a time scale on the order of 0.05 s. Specifically, projected subbeams (derived from the RT Plan as a function of time) do not show perfect temporal alignment with observed irradiated diode doses vs. time. This presents a challenge for measurement guidance. See Fig. 2 for examples of synchronization issues.

##### A.1.6 Threading effect and measurement locations

Dose in regions radiating outward from the tomotherapy isocenter is progressively harder to model due to the a “threading effect” of divergent fan beams rotating in a helix (compare to Kissick et al.^(16)^). There is high sensitivity to Y jaw accuracy and penumbra modeling, for instance. The AC detector elements are located in the periphery, 104 mm from the isocenter, and dose distributions at that location are hard to model accurately from first principles. (This also holds true for the tomotherapy primary treatment planning system (TPS), as will be shown later in this paper).

#### A.2 The algorithm

The TPDP method developed in this work attempts to address the challenges enumerated above with a number of tomotherapy‐specific modifications to the conventional ACPDP method. Because that method has been already published,^(8)^ we will describe only the steps that are new or modified to support tomotherapy. These steps are highlighted in Fig. 1.

**Figure 3:**
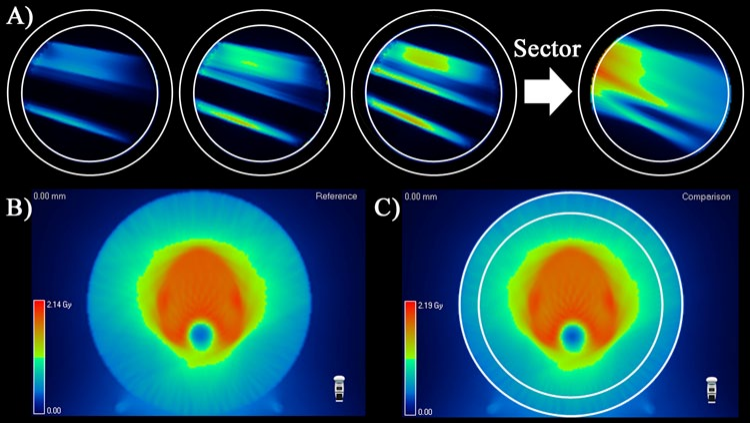
Three high spatial resolution subbeams (a) (seen on the axial cross section through the middle of the AC phantom) that are part of the group binned and added into a single “sector” which limits the impact of inevitable synchronization errors due to differences in actual vs. nominal tomotherapy MLC and gantry dynamics. TPS cumulative dose (b) for AC phantom. Tomo‐PDP “virtual gel” cumulative dose (c) after all sectors are morphed to match measurements and then summed and postprocessed, resulting in a volumetric cumulative dose to compare to the TPS‐calculated dose. (Note: In panels (a) and (c), the AC exterior and detector surface cross section are highlighted in white; in 3DVH, dose is reconstructed inside the detector surface and outside is left equal to the TPS.)

1. Synchronization of RT Plan to time‐resolved measurement points. The RT Plan's control points have cumulative meterset values in units of time rather than monitor units, so synchronization of the control points to the time‐resolved measurements is, at least at the surface, conceptually simple. Both are on an absolute time scale, and those two time scales simply need to be registered to each other. However, using the first “beam on” update from the measurement and mapping this to the start of beam delivery (i.e., first control point before cumulative meterset changes to the next) is not sufficient for either of two reasons: 1) for the first projection, MLC leaves start in the closed position, exposing no diodes, or 2) the first MLC segments may be too small to directly expose any diodes. Either reason could cause a failure to meet threshold diode current that signals the “beam on” condition and introduce time inaccuracy; however, this method is used for the first pass to get within 0.5 s accuracy.
  To refine the synchronization, dose as a function of time is calculated for key diodes (moderate cumulative dose of at least ∼50% of the maximum) using the sinogram data and the 3DVH convolution algorithm, for various time shifts in increments of 0.01 s. The eventual time sync derivation is based on the time registration giving the best dose agreement between the observed and the calculated dose at the critical diodes. Some plans can exceed the length of the AC sensitive volume. Such plans must be shifted longitudinally on the phantom (a “green laser shift”) so that the first or last beamlet projects onto the active volume. This allows the AC to register the beam‐on time, at the expense of only the ROIs within the AC sensitive volume being fully evaluated. While in theory either shift direction would work, in reality the last beamlet has to end up in the active volume as an opposite shift direction would expose electronics to the direct beam. This is one of the system limitations.
2. Organization into 3D dose “sectors.” Time‐resolved (four‐dimensional, or 4D) subbeams can be calculated in 3DVH for any integral number of projections per control point interval. One projection/control point proved far too coarsely discretized (i.e., 51 projections per rotation is inadequate for accuracy in the periphery^(17)^). Other integer multiples (2, 3, etc.) of subbeams per control point interval were tested, and two subbeams/control point interval gave the best balance of accuracy and performance. As a result, there are 102 subbeams per rotation and multiple thousands of subbeams over the whole treatment, depending on the number of rotations. However, given the high speeds of the gantry and the motion of the table, even the best time synchronization (as described above and illustrated in Fig. 2) results in a diode irradiation pattern that at times is out‐of‐sync (±0.02 s) with some of the subbeams' MLC openings derived from the nominal sinogram patterns. These errors do not manifest in predictable patterns and thus cannot be accounted for mechanistically. If per subbeam measurement‐based correction (“morphing”) was performed as in VMAT AC‐PDP,^(8)^ these minor time mismatches would cause unwarranted morphing of calculated subbeam doses to fit 4D measurement patterns that do not match for any particular small time interval. Therefore, before measurement‐guided dose morphing is performed, 4D sub‐beam dose grids are grouped (i.e., summed) in two ways to lessen the effect of per‐subbeam synchronization vacillation: 1) over fixed spans in gantry angle per rotation, and 2) over identical gantry angle spans over all rotations as the table is in motion. The resulting 4D dose summations are called “sectors” (see Fig. 3). The advantage is that sector doses map nicely to entry and exit diode columns along the long axis of the AC phantom. To ensure a sufficient number of exposed diodes, the algorithm bins subbeams according to gantry angle span equivalent to three control points ($∼21^{\circ }$). It is important to remember that within a sector dose, all subbeams' dose grids were computed at their respective projection angles; the sectors serve only to group many thousands of subbeam's dose grids into a smaller set of dose grids for dose morphing to measurements, grids that are far less sensitive to small time sync imperfections.
3. Per‐sector dose morphing. For an error‐free delivery and perfect input data (i.e., sinograms, gantry vs. time), there would be no need for morphing. There would only be a need for a simple scalar adjustment to fit the high‐resolution, volumetric sector dose (in relative units at this point) to the absolute measured dose (Gy) accumulated over each sector's associated time intervals. However, measurement‐guidance implies that the high‐resolution calculations are fit to the diode doses, thus requiring dose morphing. Each sector dose is morphed in a way similar to what conventional ACPDP uses for each subbeam, the difference being that the morphing technique is applied not to a single subbeam, but to all that contributed to that sector. Each sector has natural entry and exit dose surface (see Fig. 3) between which correction factors can be interpolated. In addition, a “loop morphing” is one along the IEC table Y direction to ensure that if any errors are uniform over an AC axial loop (i.e., specific to a longitudinal position) that the slab of dose straddling that loop is scaled accordingly. Normally this is a very minor correction.
4. Summation of 3D sector doses. After all three‐dimensional (3D) sector doses are morphed and simultaneously calibrated as absolute, high‐resolution dose grids, they are summed to give a cumulative absolute 3D phantom dose for the whole treatment.
5. Ion chamber correction of 3D phantom dose. The AC diodes are situated exclusively in the radial periphery of the phantom, with detectors displaced by ∼10.4 cm from the isocenter. This is precisely where the threading effect of helical tomotherapy is most difficult to model and creates implicit challenges for measurement guidance. To help allay such dependencies, the TPDP algorithm includes the ability to further correct (morph) a volumetric dose based on central ion chamber (IC) measurement. The AC can be fit with a “MultiPlug” insert that allows 25 fixed measurement options relative to the plug, and the plug can be rotated to produce many more options. Software helps guide the user to high‐dose, low gradient points which serve as the best measurement options, in that they are less susceptible to positioning or volume averaging errors. Upon finishing dose reconstruction using the diodes, the user can specify a measurement position on the central axial plane along with the absolute measured dose and IC active volume. If the IC‐measured dose is different from the TPDP estimate, then all dose voxels inside a longitudinal cylinder of radius, r, will be multiplied by the same IC/TPDP dose ratio. This radius, r, is defined as the radial distance from the phantom center to the measurement point. From the measurement point's radius to the detector radius, correction values are tapered down linearly to be exactly 1.00 at the detector surface to ensure the dose exactly matches measurements at the detector cylindrical surface.

### B. Validation of the algorithm

#### B.1 Static dose calculations and phantom density

Standard TomoTherapy treatment planning system (TPS) software does not allow dose calculations with the static table. While it is not necessary for routine use of the system, the ability to perform basic dose calculations with static gantry and rotational beams without additional complications of helical delivery is helpful for dosimeter calibration and evaluation. To that end, we employed an Accuray standalone dose calculator similar to that described by Kissick et al.^(16)^ It allows dose calculation for any delivery plan, whether generated through the normal treatment planning process (helical) or manually. To verify that the beam models were identical in the clinical and standalone dose calculator software, three helical plans with different jaw settings (1, 2.5, and 5 cm) were developed in the clinical software, transferred to the dose calculator and recalculated. The corresponding dose distributions were indistinguishable at the 1%/1 mm level.

Both diode arrays used in this work are embedded in polymethylmetacrilate (PMMA) phantoms. In theory, relative electron density should be used to scale the physical dimensions of the phantoms in dose calculations.^(18)^ However, the TomoTherapy TPS uses the CT number to mass density conversion tables for inhomogeneity corrections. Furthermore, those tables are not employed directly but rather used as a basis to derive tissue‐specific mass energy absorption coefficients.^(19)^ Under these circumstances, it is not clear what value should be best used to represent the PMMA and it was prudent to derive it empirically. To that end, static beams were projected on a homogeneous AC phantom and the dose was calculated with different assigned phantom densities ranging from 1.15 (relative electron density of PMMA) to 1.19 (mass density). Relative dose was compared to ionization ratios in a PMMA phantom mimicking the AC dimensions, but accommodating an 0.06 cm^3^ A1SL (Standard Imaging, Middleton, WI) ion chamber.^(15)^ Comparisons were made for static vertical beams at the locations of the entrance and exit diodes (2.9 cm from the surface) and at the center. The dose profiles were sampled along the table movement to find the maximum signal. The radiological distance between the exit and entrance measurement points is approximately 23.7 cm. The ratios were obtained for a number of field sizes ranging from 5×40 to $1\times 3.75\;{\text{cm}}^{2}$. For the 1 cm jaw setting, the entrance measurements with the A1SL chamber are unreliable due to the volume‐averaging effect. However center‐to‐exit comparisons were performed as the field size increased with beam divergence.

#### B.2 Helical array calibration

There are two valid ways to perform absolute calibration of the AC array. The manufacturer recommendation for TomoTherapy is to use an ion chamber with absolute calibration in dose to water in a flat Virtual Water phantom (Standard Imaging Inc.). The chamber is placed at the same distance from the source as the reference diode would be with the AC centered on the green lasers (74.6 cm) with the buildup equivalent to 2.9 cm of PMMA, which equals 3.3 cm of water‐equivalent plastic. A vertical (gantry at 0°) static $5\times 40\;{\text{cm}}^{2}$ beam is used. The A1SL chamber correction for beam quality $k_{q}$ is at most 0.2%^(20)^ and is customarily ignored.

An alternative method, easily implemented for C‐arm accelerators,^(15)^ is based on the premise that the TPS should be capable of an accurate dose calculation in a $10\times 10\;{\text{cm}}^{2}$ beam projected on a large, homogeneous cylindrical phantom. With the standalone dose calculator, the reference diode dose could also be determined through the static beam calculations. Both approaches were executed with the reference dose differing by no more than 1%. The calculational value was used.

The daily correction (cross‐calibration) procedure^(15)^ was performed before every set of AC measurements. A rotational $5\times 40\;{\text{cm}}^{2}$ beam with a static table was calculated on the AC phantom. The irradiation time was 48 s and included four full gantry rotations. The standalone dose calculator can export DICOM RT dose object but not the DICOM RT plan derived from the static treatment procedure defined at the operator console, outside of the clinical TPS. Angular corrections implicit in the AC software cannot be applied in absence of an RT Plan for TomoTherapy so, for these test data, they were applied manually. If the beam aperture is constant and the number of gantry rotations is whole, the average dose‐weighted angular correction factor can be applied equally to every diode without any approximation. After this factor (0.982) was applied, the measured dose in the central portion of the field was compared to the TPS, and a daily correction factor was introduced. This correction brought the median difference between the measured and calculated doses to zero. It must be noted that in essence this daily correction procedure renders the absolute calibration moot; it is an array equivalent of the daily ion chamber cross‐calibration procedure, as it is irradiated in a simple phantom and reference geometry to obtain a correction factor that takes into account the daily accelerator output variations.

The important difference between the conventional and tomotherapy dose acquisition modes is the application of certain AC correction factors. The field size correction is ignored because it is negligible for the small fields (<5 cm) and has not yet been studied for the “very small” (<1.5 cm)^(21)^ ones. The angular correction for tomotherapy measurements was introduced in 2014 (SNC Patient v. 6.5). Since the virtual inclinometer^(15)^ does not perform well with tomotherapy, the gantry angle is derived from the gantry positions and the elapsed irradiation times known from the RT Plan's control points.

#### B.3 Test plans

One of the main challenges to overcome with TPDP is the limited number of diodes irradiated by each beamlet. This issue is clearly exacerbated as the jaw width is decreased. In this study we use the two smaller jaw settings: 2.5 cm and 1 cm. The former is by far the most frequently used in our practice, while the latter is seldom used but presents a good stress test of the system. In total, we designed 19 test plans, planned and delivered with both 2.5 and 1 cm jaw settings. All cases were planned with pitch values conforming to the original 0.860/N formula.^(16)^ One of the plans turned out to be undeliverable with 1 cm jaws, so only 18 test plans were analyzed for this jaw setting. The 19 plans are described below.

##### B.3.1 Cylindrical targets

Four types of cylindrical plans were developed. First, cylindrical targets of 2.5, 5, and 7.5 cm diameter and 10 cm long were planned to receive a uniform 2.0 Gy dose per fraction. The fourth plan in the cylinder series delivered a uniform 2.0 Gy dose to a 1 cm thick cylindrical shell encompassing the AC diodes (10.4 cm from the isocenter). This plan resulted in a slowly varying, relatively uniform dose inside the shell, suitable for ion chamber measurements. We will call this case a 21 cm cylinder. Pitch values of 0.287 were used for these plans.

##### B.3.2 TG‐119 plans

The second set of plans was comprised of four cases from TG‐119^(22)^ (C‐shape, head and neck, multitarget, and prostate), each planned according to the Report. Two sets of plans were made, one using pitch 0.286 and the other using loose pitch of 0.86, for a total of eight TG‐119 test plans.

##### B.3.3 Clinical plans

Seven clinical plans were selected. Those were comprised of three head and neck sites (including two plans with two dose levels and a reirradiation plan), one gynecological pelvic plan (endometrial adenocarcinoma), one anal site with the primary target and lymph nodes irradiated to different doses, one abdominal (gallbladder), and one brain case. All clinical plans delivered 1.8 or 2.0 Gy per fraction to the primary target. Pitch values of 0.287 were used for these plans.

All plans were calculated on a “fine” TomoTherapy TPS dose grid, which translates into a voxel size between approximately 2.0 and 2.7 mm. Two dynamic quality assurance (DQA) procedures were calculated for each plan: one on the ArcCHECK and one on the Delta^4^ (ScandiDos AB, Uppsala, Sweden) phantom. For both, the RT Plan and RT Dose objects were exported for analysis as required.

All plans were delivered on the TomoTherapy machine running Hi·Art system software v. 4.2. Ours is a “hybrid” unit in that it has an older style Siemens linac coupled with an upgraded, fixed target.

#### B.4 Comparisons to the ion chamber

The ion chamber (IC) comparisons were performed on the PMMA AC phantom. The A1SL chamber was cross‐calibrated against calculated dose in the center of phantom produced by a rotational, open $5\times 40\;{\text{cm}}^{2}$ beam with the static table. It was verified that the absolute IC dose agreed with the similar calculation near the center of a 30 cm diameter Virtual Water (“Cheese”) phantom to within 1%, essentially confirming the calibration comparison results. After cross‐calibration, the test readings were taken with the MultiPlug inserted into the AC phantom, at the locations suggested by the 3DVH software as high‐dose, low‐gradient. Of 37 measurements, 10 required chamber movement to the off‐center position. The ion chamber dose was compared to the TPS and 3DVH reconstruction without the IC normalization. The calculated/reconstructed values were reported as a mean dose to a 0.057cm^3^ sphere surrounding the point of interest, approximating the ion chamber volume. The same IC data were used as inputs to the optional 3DVH IC correction.

#### B.5 ArcCHECK dose analysis

The AC time‐course measurement is used by TPDP, but the cumulative diode doses measured on the cylindrical surface can be also directly compared to the dose values extracted from TPS‐calculated dose grid. Gamma analysis^(2)^ was employed to quantify the agreement between the dose distributions. Four sets of analysis criteria were used: 3%/3 mm with global (G) and local (L) dose‐error normalization, and the same for 2%/2 mm. All dose analyses were in absolute dose. The lower dose cutoff threshold was set at 10% of the maximum dose. In addition, for each case the median dose difference between the diodes and the TPS was correlated to the difference between the IC and the TPS.

#### B.6 Comparisons to a biplanar array dosimeter

##### B.6.1 Delta^4^ description, calibration and daily correction

After the point dose comparisons with an ion chamber, the next step in TPDP validation is comparing the reconstructed volumetric dose with the biplanar absolute dosimeter, the Delta^4^. As applied to TomoTherapy, the Delta^4^ system was validated^(14)^ and used for clinical patient QA in a large longitudinal series of 264 patient plans.^(23)^ It is important to note that in both papers, gamma analysis exclusively used 3% dose‐error (global normalization)/3 mm distance criteria. The Delta^4^ dosimeter has two planar diode arrays arranged at a right angle. The detector spacing varies from 5 mm in the center of the $20\times 20\;{\text{cm}}^{2}$ active area to 10 mm on the periphery. In its basic implementation, the measured dose at the detector positions is compared to the planned dose (on the Delta^4^ 22 cm diameter cylindrical PMMA phantom) extracted from the DICOM RT Dose object transmitted from the TPS. The TomoTherapy measurement mode has two important differences from the C‐arm linac.^(14)^ First, no electrical trigger pulse is available from the accelerator and dose acquisition is triggered by sensing the radiation pulse itself. Second, the gantry angle as a function of time is unknown and, therefore, an average angular correction is applied to every diode, as opposed to the varying angle‐specific series.

The reference dose for the Delta^4^ absolute calibration was obtained in a flat solid water phantom in the same fashion as for the AC. The only difference is that the calibration geometry requires the IC to be positioned at the isocenter (85 cm from the source) and the required buildup is 5.1 cm of solid water. The daily cross‐calibration procedure is also essentially the same. The rotational $5\times 40\;{\text{cm}}^{2}$ beam with the static table was calculated on the Delta^4^ phantom, and the differences between measurements and calculations in the central portion of the field were minimized by the application of the multiplicative daily correction factor, which was then used for the subsequent measurements.

##### B.6.2 specific tests

First, Delta^4^ diode measurements were compared to the TPS in a usual fashion, using the y‐analysis tools in Delta^4^ software (May 2014 version). Second, a TPDP DICOM RT Dose grid reconstructed on the Delta^4^ phantom was imported in the Delta^4^ software as a reference dose. Although the Delta^4^ and ArcCHECK phantoms are similar in cross‐sectional shape, the TPDP dose had to be reconstructed using the patient dose pathway in 3DVH because the two phantoms differ in diameter. After spatial alignment, the differences between the measured and TPDP doses can be analyzed using the Delta^4^ software tools. For reference, TPDP was also compared to the TPS dose in 3DVH software, with the same four combinations of gamma analysis criteria.

#### B.7 Statistical analysis

Statistical analyses were performed with GraphPad Prism software (v. 6.0, GraphPad Software Inc., La Jolla, CA). Various tests were performed and the specifics of each one are provided along with the results, making the details easier to follow. If all the data series in a particular group passed the D'Augostino & Pearson normality test, the tests assuming Gaussian distribution of the data were selected. Otherwise, nonparametric statistical tests were employed. All ± errors quoted in the text correspond to one standard deviation.

## III. RESULTS

### A. Phantom density

A relative density of 1.17 assigned to PMMA in the TPS produced the overall best agreement between the calculated and measured dose ratios. This value falls in between the PMMA relative electron and mass densities. When calculations were performed with this phantom density, excellent^(24)^ percent depth dose agreement between measured and calculated data was observed for a range of field sizes. The maximum difference was 1.2% of the local dose, at a substantial depth of 23.7 cm (Table 1). Based on these results, the uniform relative density of 1.17 was assigned in the TomoTherapy TPS to both the ArcCHECK and Delta^4^ phantoms.

**Table 1:** Percent differences (local) between calculated and ion chamber (IC) measured Exit to Entrance and Exit to Center dose ratios for the various size static beams on the ArcCHECK phantom. Entrance and Exit points are 2.9 cm from the surface of the phantom

*Field Size* $Y\times X(cm^{2})$	*Difference Calculated – IC (%)*	
	*Exit/Entrance Ratio*	*Exit/Center Ratio*
5×40	0.0	0.4
5×3.75	1.0	0.5
2.5×6.25	1.2	1.1
2.5×3.75	1.2	1.2
1×6.25	–	−0.3
1×3.75	–	0.6

### B. Comparisons to the ion chamber

The descriptive statistics of the ion chamber comparisons to both the TPS and TPDP are presented in Table 2. The means of the columns corresponding to the two different jaw settings were evaluated for statistically significant differences using the paired *t*‐test. While the TPS and IC agreed well for the 2.5 cm jaws, the 3.5% difference in the high‐dose, low‐gradient areas for the 1 cm jaws was a significant fnding.^(22)^ Not surprisingly, the *t*‐test results were highly significant (p<0.0001). On the other hand, TPDP (obviously without the IC correction, in this case), shows approximately the same average difference of about 1% for both 1 and 2.5 cm jaws.

This TPDP difference between the two jaw settings was not statistically significant, indicating that, in both cases, the central point dose is on average reconstructed reasonably accurately from the peripheral diodes readings. Individual TPDP vs. IC comparisons span the range from −0.7% to +3.0%, compared to a range of −7.1% to 1.7% for TPS vs. IC.

**Table 2:** Descriptive statistics of the percent ion chamber dose difference from the TPS and TPDP (without chamber correction). N=19 for 2.5 cm jaw plans and N=18 for 1 cm plans. The means of columns with different jaws settings were compared with paired *t*‐test. Note: TPDP with chamber corrections are, by definition, 0%

	*Difference TPS–IC (%)*		*Difference TPDP–IC (%)*	
	*Jaws 1 cm*	*Jaws 2.5 cm*	*Jaws 1 cm*	*Jaws 2.5 cm*
Mean	−3.5	−0.3	1.1	1.0
Std. Deviation	1.5	1.4	1.1	1.1
Min.	−7.1	−3.1	−0.7	−0.7
Max.	−0.7	1.7	3.0	2.7
Lower 95% CI	−4.2	−1.0	0.6	0.5
Upper 95% CI	−2.7	0.4	1.6	1.5
Means different (p‐value)	Y(<0.0001)		N(0.7)	

### C. ArcCHECK dose analysis

The statistics of the γ‐analysis of the AC vs. TPS dose are presented in Table 3. Due to the poor agreement at the 3%/3 mm level, the data for 2%/2 mm are omitted. While the disagreement for the 1 cm jaws could be expected due to the previously described IC data bias, the lack of agreement for even the 3% G/3 mm criteria combination was a significant finding for the 2.5 cm jaws setting.

The possible correlation between TPS–IC dose difference and median TPS–AC difference was studied with the nonparametric Spearman test. Spearman correlation coefficients were 0.61 for the 1 cm jaws (p=0.007) and 0.51 (p=0.027) for the 2.5 cm jaws. The correlation between the peripheral and central doses is present but is not perfect, reflecting the fact that multiple factors contribute to the median TPS–AC difference.

**Table 3:** Descriptive statistics of the γ‐analysis percent passing rates, TPS vs. ArcCHECK

	*1 cm Jaws* γ *Pass Rate (%)* (N=18)		*2.5 cm Jaws* γ *Pass Rate (%)* (N=19)	
	*3% G/3 mm*	*3% L/3 mm*	*3% G/3 mm*	*3% L/3 mm*
Mean	44.6	32.4	73.9	56.6
Std. Deviation	14.7	9.0	15.2	13.6
Min.	22.1	19.6	49.7	36.3
Max.	78.0	50.2	99.7	97.4
Lower 95% CI of mean	37.2	27.9	66.5	50.0
Upper 95% CI of mean	51.9	36.9	81.2	63.1

### D. Comparisons between the TPDP, biplanar array, and TPS

In the next step, we compare TPDP dose reconstructed on the Delta^4^ “patient” (with and without the ion chamber correction) to the direct Delta^4^ biplanar measurements. The statistics for the γ‐analysis comparisons are presented in 4, 5 for the 1 and 2.5 cm jaw settings, respectively. The difference between the results corrected and uncorrected with the IC point dose were analyzed with the nonparametric Wilcoxon matched‐pairs signed‐rank test. For the cases planned with the 1 cm jaws, the IC correction leads to a small, but statistically significant, improvement in agreement between the TPDP and Delta^4^. However, the overall agreement is not impressive for any criteria combinations more stringent than 3% G/3 mm. Only 3% G/3 mm in combination with the ion chamber correction produces the lower limit of 95% confidence interval (CI) above 90% of the Delta^4^ measurement points agreeing with TPDP. On the other hand, for the 2.5 cm jaws, the IC correction does not produce a statistically significant improvement in agreement between the TPDP and Delta^4^. However, the overall agreement is much better, with the lower limit of the 95% CI reaching the 90% agreement rate for the 2% G/2 mm criteria in combination with the IC correction. Although not reaching significance, the agreement levels improve consistently with the application of the IC correction. Therefore in the last test, the IC‐corrected results were used when comparing the TPDP and Delta^4^ dose grids to the TPS. The γ‐analysis agreement rates are presented side by side for the TPDP vs. TPS and Delat^4^ vs. TPS comparisons. The data for the 2.5 cm jaws setting are provided in Table 6. Because the 1 cm jaw plans showed a large mean disagreement between the calculated and measured point doses in the high‐dose low‐gradient regions, the dose distribution agreement data cannot be meaningfully discussed. The results of the comparisons between TPDP and Delta^4^ vs. the TPS are mixed. Depending on the criteria, the differences range from nonsignificant to highly significant based on the Wilcoxon test (Table 6). The smallest difference in mean passing rates is observed for the 3% G/3 mm criteria (0.9% in favor of the Delta^4^), while the largest one is 13% for the 2% L/2 mm analysis. The larger differences in the passing rates are associated with the local dose error normalization, indicating that the differences between the TPDP and the Delta^4^ tend to occur more in the lower dose region.

**Table 4:** Descriptive statistics of the γ‐analysis passing rates with different criteria combination, comparing TPDP with and without IC correction to the Delta^4^ measurements for the 1 cm jaw plans (N=18). Median dose difference: No IC: −1.3%±1.0%; IC: −0.8%±1.0%

	γ‐*Analysis Pass Rate, TPDP vs. Delta^4^ (%)*							
	*3% G/3 mm*		*3% L/3 mm*		*2% G/2 mm*		*2% L/2 mm*	
	NO IC	IC	NO IC	IC	NO IC	IC	NO IC	IC
Mean	91.8	95.2	83.0	87.1	78.3	83.8	67.5	75.1
Std. Dev.	8.4	6.7	11.8	10.2	13.7	12.0	14.6	13.5
Min.	74.3	76.7	64.8	66.9	55.1	59.8	47.4	51.9
Max.	100.0	100.0	99.2	99.2	97.4	99.5	94.1	95.2
Lower 95% CI of mean	87.7	91.9	77.2	82.0	71.5	77.8	60.3	68.4
Upper 95% CI of mean	96.0	98.6	88.9	92.1	85.1	89.8	74.7	81.8
Means different (p‐value)	Y(0.005)		Y(0.008)		Y(0.002)		Y(<0.001)	

**Table 5:** Same as Table 4, but for 2.5 cm jaws (N=19). Median dose difference: No IC: −0.6%±0.6%; IC: −0.3%±0.8%

	γ*‐Analysis Pass Rate, TPDP vs. Delta^4^ (%)*							
	*3% G/3 mm*		*3% L/3 mm*		*2% G/2 mm*		*2% L/2 mm*	
	*NO IC*	*IC*	*NO IC*	*IC*	*NO IC*	*IC*	*NO IC*	*IC*
Mean	98.8	99.1	95.0	95.7	91.6	93.4	84.1	86.0
Std. Dev.	1.5	1.8	3.9	4.8	4.2	7.1	7.9	9.0
Min.	93.9	92.3	87.1	79.5	84.3	68.4	71.6	57.6
Max.	100.0	100.0	100.0	100.0	99.9	99.9	96.8	98.9
Lower 95% CI of mean	98.1	98.3	93.1	93.4	89.6	90.0	80.3	81.6
Upper 95% CI of mean	99.6	100.0	96.8	98.0	93.6	96.8	87.9	90.3
Means different (p‐value)	N(0.3)		Y(0.037)		N (0.07)		N(0.13)	

**Table 6:** Descriptive statistics of the γ‐analysis passing rates with different criteria combination, comparing TPS vs. TPDP and TPS vs. Delta^4^ for the 2.5 cm jaw plans (N=19). Median dose difference: TPS vs. TPDP: 4.4%±1.7%; TPS vs. Delta^4^: 0.6%±0.6%

	γ*‐Analysis Pass Rate, TPS vs. TPDP, TPS vs. Delta^4^ (%)*							
	*3% G/3 mm*		*3% L/3 mm*		*2% G/2 mm*		*2% L/2 mm*	
	*TPDP*	*Delta^4^*	*TPDP*	*Delta^4^*	*TPDP*	*Delta^4^*	*TPDP*	*Delta^4^*
Mean	98.1	99.0	86.0	93.4	90.7	92.8	69.0	82.0
Std. Dev.	2.6	2.6	7.4	6.7	9.3	6.5	12.2	10.2
Min.	91.1	88.7	73.6	68.5	62.4	73.1	50.8	49.6
Max.	100.0	100.0	99.8	99.7	99.2	99.9	95.1	98.1
Lower 95% CI of mean	96.9	97.8	82.4	90.2	86.2	89.7	63.2	77.0
Upper 95% CI of mean	99.4	100.0	89.5	96.6	95.2	95.9	74.9	86.9
Means different (p‐value)	Y (0.03)		Y(0.0004)		N(0.16)		Y(<0.00001)	

## IV. DISCUSSION

### A. Comparisons to the ion chamber

While for the 2.5 cm jaws the average difference between the TPS and IC was minimal (−0.3%±1.4%), the result for the 1 cm jaw (−3.5%±1.5%) was clearly outside of the generally accepted ±1.5% range demonstrated in TG‐119.^(22)^ In an attempt to find the immediate cause of this discrepancy, we turned to the individual static beam measurements. Comparison of the measured (IC) and calculated dose at the center of a cylindrical phantom demonstrated that for the 1 cm jaw settings the measured dose was consistently 5.5% to 6% higher than the TPS prediction, depending on the number of the open MLC leaves. Since the IC dose is higher than the TPS, the discrepancy cannot be blamed on the chamber volume averaging effect in a narrow beam. ArcCHECK diode readings at the beam entrance agreed with the IC dose at the phantom center. Apparently the modulated helical delivery dynamics reduce the error to 3.5% on average, but the significant bias remains. As mentioned in the TG‐148 Report,^(20)^ in the course of routine QA the user does not have access to the calculated static beam dose distribution to compare to the IC reading.

We hypothesized that with a loose pitch the results might be closer to the static beam. Comparison between the four TG‐119 plans optimized with 0.287 and 0.860 pitch revealed a small difference in the average TPS–IC difference, −3.4%±1.5% vs. −4.1%±1.0%. While the direction of the change followed the hypothesis, it is not statistically significant and is not likely to be of practically importance. Unlike the TPS, the TPDP–IC differences are essentially the same for both jaw settings. The mean error does not exceed 1.1%±1.1%, which implies that on average the impact of the IC correction on the TPDP results should be moderate.

It must be noted that the 1 cm jaws setting is very seldom used in our clinical practice. When it was used, the plans showed better than 90% γ(3% G/3 mm) passing rates in comparison with the Delta^4^. Moreover, in the current work 14 of 19 plans exhibited >95% agreement rate between the TPS and Delta^4^. This is yet another example of how the 3%/3 mm criteria may hide a systematic commissioning error.^(25)^ The possible remedy to eliminate the dose bias is under investigation. While this dose bias is of clinical concern, it is, ironically, beneficial for the current study, as it provides a built‐in test of TPDP's sensitivity to dose errors.

### B. ArcCHECK dose analysis

The agreement between the TPS and AC diode readings is poor regardless of the jaw setting (Table 3). Since our results disagreed with a previous report by Bresciani et al.,^(26)^ they were spot‐checked with a second ArcCHECK unit. While there were expected minor differences in the measured datasets, the trend of the TPS substantially underestimating the peripheral dose remained intact. The combination of our results with those previously reported^(26)^ suggest that the peripheral dose is sensitive to the potential differences that are machine‐specific (e.g., in the machine Y jaw alignment). The median differences between the TPS and the diode readings for all detectors above the 10% threshold were 12.1%±4.0% and 7.8%±2.2% for the 1 cm and 2.5 cm jaw settings, respectively. The difference in the median values for the two jaws settings (4.3%) is in line with the ion chamber central dose differences (3.2%). This suggests that the AC response is not noticeably affected by the field size, despite potential partial loss of the charged particles equilibrium in the 1 cm beam.

While the differences between our results and the previous report are noteworthy, they are not entirely unexpected. It was described previously how a small change in the arc aperture can lead to a substantial variation of the peripheral (ArcCHECK) dose for the conventional arcs.^(27)^ The situation is even more complicated in tomotherapy, as dosimetric effects of the small disagreements between the calculated and delivered fluence in the penumbra of the diverging helical beam on the periphery of the phantom have not been studied at all.

**Figure 4:**
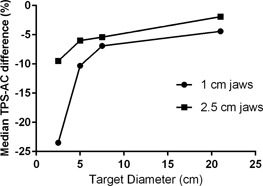
Median percent dose difference between the TPS and ArcCHECK for cylindrical targets of varying diameter, with 1 and 2.5 cm jaws.

Just as with conventional arcs,^(27)^ the difference in the peripheral dose between the TPS and AC is strongly dependent on the beam aperture width. Figure 4 shows how the median difference between the calculated and measured (AC) TomoTherapy dose difference increases with the decrease in the cylindrical target diameter. For the 1 cm jaws, as the target diameter is reduced from 21 to 2.5 cm, the median dose difference increases from −4.4% to −23.5%, or more than fivefold. For the 2.5 jaws the change is less dramatic, but is still very clear (Fig. 4). These consistent patterns of change further confirm that the observed differences are real and not the measurement artifact. As the target diameter decreases, the AC diodes location becomes “more peripheral” in that the accuracy of the dose calculation there becomes more and more dependent on the exact representation of the penumbra and out of field portions of the dose profile, which are typically the weakest points of any TPS beam model.

There are also subtle, but important, details that should be factored into comparing the current ArcCHECK results with Bresciani et al.^(26)^ First, the authors did not specify how the AC was calibrated and there are no ion chamber data to make an inference. In addition, it was assumed that an angular correction was applied to the diode readings. Such correction was not available for tomotherapy AC measurements in 2013, leading to the average measurement dose overestimation by about 1.8%. Unless the cross‐calibration against the TPS was used, this could potentially substantially alter the passing rates with 1% or 2% dose‐error criteria, and somewhat affect those using 3%. Finally, it is not specified if the “measurement uncertainty” feature in the AC software was turned on, as it was in TG‐119^(22)^ for the planar array. If so, it effectively increases the dose‐difference threshold by ∼1%, leading to the artificially inflated agreement rates.

We also highlight an important caution: it might be tempting to use the plan‐class–specific methodology^(28)^ for cross‐calibrating the AC against the TPS in a “simple” helical field. However, if a standard field described in TG‐148^(20)^ delivering a uniform dose to a 8 cm diameter cylinder is used verbatim, the calibration of the AC can be altered greatly. To illustrate this point, we “cross‐calibrated” the AC, introducing a single multiplicative correction factor that maximized agreement with the TPS for the 7.5 cm diameter cylinder. The resulting average TPS vs. AC agreement rate for the set of TG‐119 and clinical plans with 2.5 cm jaw increased from 86.7%±10.8% to 94.2%±5.9%. This shows that the concept of the plan‐class–specific correction factor should be applied with caution in the peripheral areas, where the TPS may be less accurate. Developing correction factors based on helical delivery can lead to vastly different results from the static‐table calibration/correction. While the passing rates with plan‐class–specific cross‐calibration may be higher, they would hide the true difference between the TPS calculations and measurements on the periphery.

Regardless of the potential methodology differences, the disagreement with the previously published data served a useful purpose, as it led to the realization that the same relationship between the peripheral and central dose cannot be assumed across all tomotherapy systems. As a result, the IC correction technique was developed to mitigate any potential differences, given that TPDP uses a standard dose model for all tomotherapy machines, though clearly for our test data, the required extra correction was modest.

### C. Comparisons between the TPDP, biplanar array, and TPS

For these studies on our TomoTherapy unit, the IC correction leads to a modest improvement to the agreement between the TPDP and Delta^4^. The effect is larger for the 1 cm jaws, consistent with the notion that the narrow field exposes fewer diodes to the high dose at any given time, thus complicating the MGDR process. For the 2.5 cm jaws, the IC‐corrected TPDP dose distributions compare favorably with the Delta^4^ at the 3% G/3 mm level (99.1%±1.8%). The average passing rates get progressively lower as the analysis criteria tighten up (Table 5). With the lower 95% confidence interval (CI) of mean ≥90% as a cutoff point, the agreement can be considered satisfactory at the 3% L/3 mm and 2% G/2 mm levels. With 2% L/2 mm criteria the agreement is suboptimal. This is in contrast to the VMAT data, which showed over 95% agreement rates between 3DVH and the Delta^4^ at the 2% L/2 mm level for the three TG‐119 datasets. We attribute this difference to the necessary approximations introduced in the tomotherapy version of MGDR compared to VMAT, as described in the Methods section above. Dose reconstruction is even more challenging with the 1 cm jaw setting. As a result, the lower 95% CI of the mean agreement rate between the TPDP and Delta^4^ is above 90% only for γ(3% G/3 mm). Therefore, for the jaw setting of 1 cm, we cannot ascertain that the QA results using TPDP would be meaningful with the criteria tighter than 3% G/3 mm.

While this level of accuracy may be suboptimal relative to MGDR for IMRT and VMAT,^(25)^ the 3% G/3 mm evaluation criteria are still widely employed and, with the exception of Bresciani et al.,^(26)^ were used in every publication dealing with TomoTherapy dosimetric accuracy. Specifically, the known reports of the Delta^4^ validation and use with tomotherapy were based on the γ(3% G/3 mm) criteria. This is perhaps a refection of the more general problem that it is hard to find a dosimeter to reliably sample tomotherapy dose distribution with, say, 2% L/2 mm accuracy level. This affects the precision of both new dosimeter validation and TPS dose verification. Great care must be taken to achieve even 3% dose readout accuracy with radiochromic film.^(29)^ Planar electronic arrays suffer from uncorrected angular dependence reducing their accuracy.^(30)^ A chamber‐based array with a phantom cavity partially compensating for the angular dependence could be successfully compared to the TomoTherapy TPS at the 5%/3 mm level in one publication^(31)^ and 3%/3 mm in another.^(32)^ Another planar chamber array required up to 3% plan‐specific correction factor to achieve acceptable agreement with the TPS, again at the 3% G/3 mm level.^(33)^ This was attributed to the “uncertainties of TomoTherapy delivery”. Chamber‐based arrays have limited spatial resolution, reducing their usefulness in the high gradient areas.^(34)^ Volumetric radiochromic plastic or gel dosimeters which provide the most comprehensive way of evaluating 3D dose reconstruction are typically used with global 3% dose‐error criteria.^9^, ^35^ The AAPM Report on the QA for helical tomotherapy (TG‐148)^(20)^ recommendation for evaluating agreement between calculated and measured TomoTherapy dose distribution is ≥90% γ(3% G/3 mm) passing rate for all plans. Based on the ion chamber and Delta^4^ results in the current work, years of patient‐specific QA experience, and the results of the RPC end‐to‐end test on the anthropomorphic head phantom,^(36)^ we believe that the studied TomoTherapy unit is commissioned in accordance with the current standard of practice, excluding the 1 cm jaw setting that needs corrective action. Supporting this assertion is the fact that the lowest γ(3% G/3 mm) agreement rates between TPDP or Delta^4^ and the TPS was 91.1% and 88.7%, respectively, with the means of 19 cases at 98.1%±2.6% and 99%±2.6%, respectively (Table 6). This is in stark contrast to the low passing rates — for any reasonable criteria combination — if one compares TPS to ArcCHECK diodes alone (Table 3).

**Figure 5:**
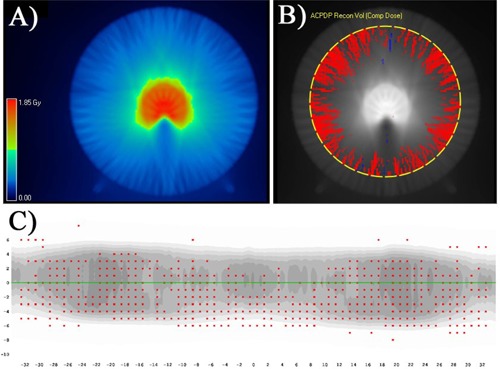
Central axial plane (a) for the TG‐119 prostate, 2.5 cm jaw plan, pitch 0.286. TPDP–TPS dose differences (b) (red denotes TPDP > TPS by more than 2%) showing TPS calculations low in the periphery but within 1% centrally. This was a typical error pattern and is consistent with the very low TPS vs. ArcCHECK diode passing rates (c), but higher passing rates for the same TPS dose grids compared to TPDP or Delta^4^, both of which are not limited to sampling peripheral dose. Red dots (c) correspond to the diodes that fail 2% G/2 mm gamma comparison against the TPS (43.9% passing rate).

Finally, as discussed in the previous section, the TPS vs. ArcCHECK (diodes only) analysis showed TPS calculations consistently low compared to the peripheral dose measured by the diode surface (radius 10.4 cm) which in turn caused low pass rates, as seen in Table 3. However, TPS vs. TPDP passing rates (Table 6) were much higher, as the TPS errors in low‐dose regions were balanced by the more central voxels. This is further evidence that the location of the detectors in a 3D phantom can have a drastic impact on passing rates, with all else equal, as has been pointed out previously.^(27)^ We illustrate this with Fig. 5. A TPS vs. TPDP difference map shows the many points different by more than 2% (local) are confined to the lower dose periphery, exactly where the diodes are. At the same time high‐dose, central regions agree well within 1%. This figure also partially illustrates why accurately calculating the dose at the diodes' locations is so hard, as the dose distribution constitutes a complex streak pattern.

Prior studies on MCPD^6^, ^7^, ^37^, ^38^ and ACPDP^8^, ^9^, ^10^, ^11^, ^25^ have shown acceptable accuracy as verified by comparison to independent dosimeters, as well as using both *in silico* simulations of known errors (with known results) and independent measurement of induced‐errors vs. the PDP estimates. At those previously established levels of accuracy, a physicist can have reasonable confidence in both the sensitivity and specificity of dose and DVH analysis using those methods. However, TPDP had to solve many challenges unique to helical tomotherapy, the most important of which were: 1) very small and complex exposed areas as a function of time, given the narrow binary leaves relative to the relatively sparse diodes at entry and exit surfaces of the measurement array; and 2) extreme dynamics of the MLC leaves and the gantry speed which cannot be effectively discretized even by 50 ms measurement updates. These challenges were handled reasonably well, but the imperfections in Tomo‐PDP could, in theory, themselves lead to “false negatives” in QA (e.g., not reporting an important error) or “false positives” (e.g., predicting an error that is not real). This is especially a concern if the region(s) of error are overlapping with target volumes and/or organ‐at‐risk volumes. So, one should keep this in mind and perhaps adopt more lenient DVH‐matching criteria due to the potential uncertainty of the dose reconstruction method itself. We do not quantify the specificity and sensitivity of TPDP in this paper. First, it would be a subject for a separate full‐length report. Second, if it were a goal, TomoTherapy is a rather closed system and inducing known errors for empirical verification would be quite difficult. Although TPDP is imperfect, it certainly highlighted some limitations of the TomoTherapy system in the ability to accurately predict the delivered dose. Other recent publications^39^, ^40^ suggest similar limitations in the TomoTherapy TPS dose calculation. These errors are worth investigating at the level of commissioning of the system and, in this regard, TPDP could be quite valuable. On a per‐patient basis, the value of TPDP is that it is measurement‐guided and generates the error map on the patient dataset; as such, any large differences are worth investigating.

## V. CONCLUSIONS

The volumetric measurement‐guided dose reconstruction algorithm required substantial modifications to accommodate the much faster delivery dynamics of the tomotherapy machines compared to the C‐arm accelerator VMAT. The necessary approximations and hardware limitations lead to the somewhat reduced reconstruction accuracy. However the volumetrically reconstructed dose agrees very well with an independent array dosimeter and TomoTherapy TPS when gamma analysis with 3% G/3 mm is used, which is consistent with the current standard of practice. The optional ion chamber‐based correction results in a modest improvement in dose agreement. When properly calibrated in a static table beam, the ArcCHECK alone samples only the peripheral dose and, at least for the studied TomoTherapy unit, can report poor agreement with the TPS for the plans that are in reality clinically acceptable.

## ACKNOWLEDGMENTS

The authors gratefully acknowledge useful discussions and help with comparison measurements by Dr. Robert Stanton (UF Health Cancer Center – Orlando Health), Dr. Jeff Kapatoes, and Mr. Amir Moghadam (Sun Nuclear Corp.). This work was supported in part by a grant from Sun Nuclear Corp.

## Supporting information

Supplementary Material
